# Super.FELT: supervised feature extraction learning using triplet loss for drug response prediction with multi-omics data

**DOI:** 10.1186/s12859-021-04146-z

**Published:** 2021-05-25

**Authors:** Sejin Park, Jihee Soh, Hyunju Lee

**Affiliations:** 1 School of Electrical Engineering and Computer Science, Gwangju Institute of Science and Technology, Gwangju, South Korea; 2 Graduate School of Artificial Intelligence, Gwangju Institute of Science and Technology, Gwangju, South Korea

**Keywords:** Precision oncology, Multi-omics data, Drug response prediction, encoder using supervised methods, Triplet loss, Pharmacogenomics

## Abstract

**Background:**

Predicting the drug response of a patient is important for precision oncology. In recent studies, multi-omics data have been used to improve the prediction accuracy of drug response. Although multi-omics data are good resources for drug response prediction, the large dimension of data tends to hinder performance improvement. In this study, we aimed to develop a new method, which can effectively reduce the large dimension of data, based on the supervised deep learning model for predicting drug response.

**Results:**

We proposed a novel method called Supervised Feature Extraction Learning using Triplet loss (Super.FELT) for drug response prediction. Super.FELT consists of three stages, namely, feature selection, feature encoding using a supervised method, and binary classification of drug response (sensitive or resistant). We used multi-omics data including mutation, copy number aberration, and gene expression, and these were obtained from cell lines [Genomics of Drug Sensitivity in Cancer (GDSC), Cancer Cell Line Encyclopedia (CCLE), and Cancer Therapeutics Response Portal (CTRP)], patient-derived tumor xenografts (PDX), and The Cancer Genome Atlas (TCGA). GDSC was used for training and cross-validation tests, and CCLE, CTRP, PDX, and TCGA were used for external validation. We performed ablation studies for the three stages and verified that the use of multi-omics data guarantees better performance of drug response prediction. Our results verified that Super.FELT outperformed the other methods at external validation on PDX and TCGA and was good at cross-validation on GDSC and external validation on CCLE and CTRP. In addition, through our experiments, we confirmed that using multi-omics data is useful for external non-cell line data.

**Conclusion:**

By separating the three stages, Super.FELT achieved better performance than the other methods. Through our results, we found that it is important to train encoders and a classifier independently, especially for external test on PDX and TCGA. Moreover, although gene expression is the most powerful data on cell line data, multi-omics promises better performance for external validation on non-cell line data than gene expression data. Source codes of Super.FELT are available at https://github.com/DMCB-GIST/Super.FELT.

**Supplementary Information:**

The online version contains supplementary material available at 10.1186/s12859-021-04146-z.

## Background

Prediction of drug response for each patient is highly important in precision oncology. However, it is quite challenging as the drug response for each patient could vary owing to genetic differences [1]. Although a cohort of patients may use the same anticancer drug, the therapeutic effect may not be the same because of multiple genetic factors [1, 2]. Thus, multi-omics data are required for precision oncology [3], and the success of precision medicine relies on effectively utilizing the combination of multi-omics data.

Recently, large-scale omics data have been made publicly accessible, including Genomics of Drug Sensitivity in Cancer (GDSC) [4], Cancer Cell Line Encyclopedia (CCLE) [5], Cancer Therapeutics Response Portal (CTRP) [6], Patient-Derived tumor Xenografts (PDX) encyclopedia [7], and The Cancer Genome Atlas (TCGA) [8]. These datasets provide multi-omics data that consists of gene expression, somatic mutation and copy number aberration (CNA), and response to multiple drugs.

The translatability of prediction models to actual patients is challenging in drug response studies [9, 10]. To achieve high performance in patients, the models for predicting drug response should be trained on in vivo data. However, the number of in vivo patient records with drug response, such as in TCGA [8], is smaller than that in in vitro datasets, such as GDSC [4]. Owing to insufficient in vivo information, a computational model for drug response prediction would require the translatability from in vitro to in vivo data. In other words, a model trained on in vitro data should have high prediction performance when tested on in vivo data.

Recently, many computational methods, such as support vector machines (SVM) [11], autoencoder (AE) [12–16], Bayesian multitask multiple kernel learning [17, 18], random forest [14, 19–21], and neural network models [22] have been proposed for predicting drug response. Especially, algorithms for dimensionality reduction, such as AE, stacked AE [16], and variational autoencoder (VAE) [23], have been frequently used. Many studies have focused on dimensionality reduction because performance of methods could largely depend on how well the required features are extracted from high-dimensional and complex multi-omics data [24]. Ding et al. [13], AutoBorutaRF [14], DeepDR [15], DeepDSC [16], and MOLI [25] are models for drug response prediction using multi-omics data.

Ding et al. [13] uses AE for selecting features, and an elastic net and SVM for classifier. In AutoBorutaRF [14], random forest is used for classifier after feature selection with AE and Boruta algorithm [26]. In DeepDR [15], AE is first pre-trained with TCGA data without drug response data, and the weights from AE are used for the initialization of a prediction model. Finally, the model is trained on the labeled GDSC data. DeepDSC [16] uses a stacked deep AE for reducing the large dimension of multi-omics data. MOLI [25] encodes multi-omics data with a deep neural network; however, its difference from other models is the integrated loss function, where encoders and a classifier are trained together for sharing each loss. The notable loss function of MOLI is triplet loss function [27]; the loss function would be appropriate for distinguishing resistant samples from sensitive samples. In MOLI, encoders using the triplet loss and a classifier were trained jointly. Nonetheless, we suspected that the performance of drug response prediction can be improved if the loss functions are trained in a different way.

In this study, we focused on how accurately an encoder can extract important features for classifying samples as drug response resistant  or sensitive. We proposed a novel method, Supervised Feature Extraction Learning using Triplet loss (Super.FELT). In Super.FELT, there are three stages, namely, feature selection, feature encoding, and classification. In the feature selection stage, Super.FELT uses variance threshold based on the elbow method to extract significant features of omics-data. In the feature encoding stage, each dataset is assigned to different encoders, and each encoder is trained using a supervised method, wherein an objective function is triplet loss function. Because the encoder is based on the supervised method, feature selection for reducing the large dimension of omics data would improve the efficiency of the encoder. Therefore, Super.FELT could extract important features from each omics data better than previous models. To assess the translatability of our proposed model, we trained Super.FELT using GDSC dataset and tested on CCLE, CTRP, PDX, and TCGA datasets. Our results showed that Super.FELT is superior to the other drug response prediction methods on external datasets.

## Methods

### Datasets

**Table 1:** Datasets used in our experiment

Type	Dataset	Usage	# of drugs	Avg of cell lines	Avg of R/S
in vitro	GDSC	Training and cross validation	243	776	685/91
in vitro	CCLE	External validation	10	224	200/24
in vitro	CTRP	External validation	62	404	340/64
in vitro	PDX	External validation	6	31	27/4
in vivo	TCGA	External validation	13	27	14/13

“#” represents the number of drugs.

“Avg” represents the average number.

“R” and “S” indicate resistant and sensitive samples, respectively

In this study, we used in vitro (GDSC, CCLE, CTRP, and PDX Encyclopedia) and in vivo (TCGA) datasets, including multi-omics data (gene expression, CNA, and mutation) and drug responses (Table 1). For GDSC, we used 243 non-duplicated drugs among 265 drugs obtained from Iorio et al. [28], which contains the binary drug response information (resistant or sensitive) for cell lines. For each drug, the numbers of cell lines are different; the average number of cell lines and resistant and sensitive samples are 776, 685, and 91, respectively (Additional file 1: Table S1). Raw gene expression data were obtained from https://www.ebi.ac.uk/arrayexpress/experiments/E-MTAB-3610/files/raw/. CNA and mutation data were downloaded from ftp://ftp.sanger.ac.uk/pub/project/cance rrxgene/releases/release-7.0/Gene_level_CN.xlsx and ftp://ftp.sanger.ac.uk/pub/project/cancerrxgene/releases/release-7.0/WES_variants.xlsx, respectively. The drug response data based on ln(IC50) values were obtained from Table S5 (C) of Iorio et al. [28] (https://www.cancerrxgene.org/gdsc1000/GDSC1000_WebResources///Data/suppData/TableS5C.xlsx).

For CCLE and CTRP datasets, we obtained datasets from PharmacoGx (R package) [29], and CNA data were downloaded from https://data.broadinstitute.org/ccle_legacy_data/dna_copy_number/CCLE_copynumber_2013-12-03.seg.txt. Because CCLE and CTRP were used as external validation, we selected non-overlapping cell lines with GDSC for existing drugs in GDSC based on Table S4 (E), (F), (I), and (J) of Iorio et al. [28]. The total numbers of drugs, average numbers of samples, and resistant and sensitive samples were 10, 224, 200, and 24 for CCLE and 62, 404, 340, and 64 for CTRP, respectively (Tables 2 and 3).

**Table 2:** Profiles of CCLE dataset

Drug	Sensitive	Resistant	Total
17-AAG	20	142	162
Crizotinib	27	294	321
Erlotinib	58	276	334
Nilotinib	17	131	148
Nutlin-3a	6	156	162
PD-0325901	50	115	165
PD-0332991	18	131	149
PHA-665752	23	297	320
PLX4720	10	150	160
Sorafenib	15	309	324

**Table 3:** Profiles of CTRP dataset

Drug	Sensitive	Resistant	Total
Afatinib	77	302	379
Axitinib	142	285	427
AZD7762	56	384	440
AZD8055	80	365	445
BI-2536	41	548	589
Bleomycin	68	316	384
BMS-345541	51	340	391
BMS-754807	48	362	410
Bortezomib	57	514	571
Bosutinib	65	357	422
CAL-101	61	345	406
Cytarabine	65	368	433
Dabrafenib	23	177	200
Dasatinib	200	377	577
Docetaxel	56	136	192
Doxorubicin	125	297	422
Etoposide	94	318	412
EX-527	17	355	372
GDC0941	120	315	435
Gefitinib	66	349	415
Gemcitabine	28	345	373
GW843682X	33	504	537
Imatinib	71	494	565
JNJ-26854165	100	283	383
KU-55933	64	361	425
Lapatinib	68	476	544
Masitinib	64	328	392
Methotrexate	16	402	418
MG-132	10	204	214
Mitomycin C	145	260	405
MK-2206	100	306	406
NVP-BEZ235	58	252	310
NVP-TAE684	182	379	561
Obatoclax Mesylate	73	346	419
OSI-027	79	331	410
OSI-930	23	304	327
PAC-1	32	347	379
Paclitaxel	147	415	562
Parthenolide	126	408	534
Pazopanib	29	377	406
PHA-793887	94	309	403
PI-103	24	375	399
PIK-93	69	328	397
Piperlongumine	41	350	391
Ruxolitinib	73	324	397
SN-38	73	259	332
SNX-2112	53	358	411
Sunitinib	60	507	567
Tamoxifen	14	372	386
Temozolomide	12	395	407
Temsirolimus	45	194	239
TG101348	65	325	390
TGX221	63	513	576
TPCA-1	36	365	401
Trametinib	26	171	197
Tubastatin A	43	170	213
TW 37	24	371	395
Vorinostat	61	360	421
VX-680	15	46	61
YK 4-279	34	398	432
YM155	65	304	369
ZSTK474	23	377	400

**Table 4:** Profiles of PDX & TCGA dataset

Dataset	Drug	Sensitive	Resistant	Total
PDX	5-Fluorouracil	1	22	23
PDX	Cetuximab	5	55	60
PDX	Erlotinib	3	18	21
PDX	Gemcitabine	7	18	25
PDX	Paclitaxel	5	38	43
PDX	Trametinib	3	16	19
TCGA	5-Fluorouracil	16	9	25
TCGA	Cetuximab	6	3	9
TCGA	Cisplatin	58	6	64
TCGA	Docetaxel	6	9	15
TCGA	Doxorubicin	4	13	17
TCGA	Erlotinib	2	2	4
TCGA	Etoposide	2	2	4
TCGA	Gemcitabine	24	37	61
TCGA	Mitomycin C	1	3	4
TCGA	Paclitaxel	24	9	33
TCGA	Sorafenib	1	13	14
TCGA	Tamoxifen	9	3	12
TCGA	Temozolomide	11	77	88

For PDX and TCGA datasets, which are also used for external validation, we performed tests for drugs having at least one resistant and sensitive samples and at least four samples in total among existing drugs in GDSC. As a result, 6 and 13 drugs were tested for PDX and TCGA, respectively, where the average numbers of samples and resistant and sensitive samples are 31, 27, and 4 for PDX and 27, 13, and 14 for TCGA (Table 4). PDX dataset is available in Supplementary File of Gao et al. [7] (https://static-content.springer.com/esm/art%3A10.1038%2Fnm.3954/MediaObjects/41591_2015_BFnm3954_MOESM10_ESM.xlsx), where “CR” and “PR” are “Sensitive”, and “SD” and “PD” are “Resistant”. Omics data of TCGA were downloaded from Firehose Broad GDAC (http://gdac.broadinstitute.org/runs/stddata__2016_01_28/). For drug response of TCGA data, we used Table S2 of Ding et al. [30], where “Complete Response” and “Partial Response” are “Sensitive”, and “Clinical Progressive Disease” and “Stable Disease” are “Resistant”.

We preprocessed each omics dataset using source codes of Sharifi-Noghabi et al. [25]. The details about data preprocessing steps are described in the Supplementary File of Sharifi-Noghabi et al. [25]. The preprocessing steps have been briefly explained as follows. For measuring gene expression, microarray Affymetrix Human Genome U219 (GDSC), HG-U133 Plus 2.0 (CCLE and CTRP), and RNA-seq on Illumina Hiseq (TCGA and PDX) were used. The expression values of GDSC were extracted from raw CEL files, and robust multi-array average (RMA) normalization was performed. For TCGA gene expression data, expression values, which were normalized by the RNA-Seq by expectation maximization (RSEM) method [31], were converted to transcripts per million (TPM) values and transformed to ${log_2 }$values. For PDX samples, fragments per kilobase of exon model per million reads mapped (FPKM) values of the downloaded gene expression data were converted to TPM [32]. Pairwise homogenization process [33] was used to remove batch effects of gene expression of CCLE, CTRP, PDX, and TCGA data based on GDSC. In copy number profiles, because copy numbers were measured by Affymetrix SNP6.0 arrays in TCGA, HapMap [34] and log2-transformed were used for normalization. In addition, the circular binary segmentation algorithm [35] was used to calculate segments, and all genes with deletions or amplification were assigned as one, and those without deletion or amplification were assigned as zero. Because PDX and GDSC provided gene-level estimated total copy numbers, copy number was converted into log2-transform. Copy number data of CCLE and CTRP were processed using the same pipeline of TCGA except for removing germline CNA. For mutation, silent mutations were filtered out, and only those affecting the protein structure were used. As CCLE and CTRP provide only 1651 genes for mutation data, we assigned no mutations for genes that are not contained in these datasets but contained in GDSC.

Because PharmacoGx package [29] only provides continuous IC50 values for CCLE and CTRP datasets, we additionally processed them into binary labels (sensitive and resistant). We used a binarization scheme employed in LOBICO [36], which was also used by Iorio et al. [28] for assigning binary labels in GDSC. First, we converted IC50 values into ln(IC50). LOBICO [36] sampled 1000 data per one cell line based on a normal distribution with their given confidence intervals. However, we fixed the confidence interval as 0.5 and sampled 100 data per one cell line because we did not have the confidence interval values for CCLE and CTRP datasets. Second, we performed kernel density estimation on the distribution of 100 ${\times }$ *n* data, where *n* is the total number of cell lines of a target drug, using a normal distribution with bandwidth 0.5 as kernel. Finally, using the method of LOBICO [36] for obtaining the population of resistant cell lines, we obtained the threshold for binary values of continuous IC50 values. Additional file 1: Tables S2, S3, S4, and S5 present ln(IC50) thresholds for deciding binary labels and binary labels assigned for each drug in CCLE and CTRP, respectively.

### Super.FELT

**Fig. 1:**
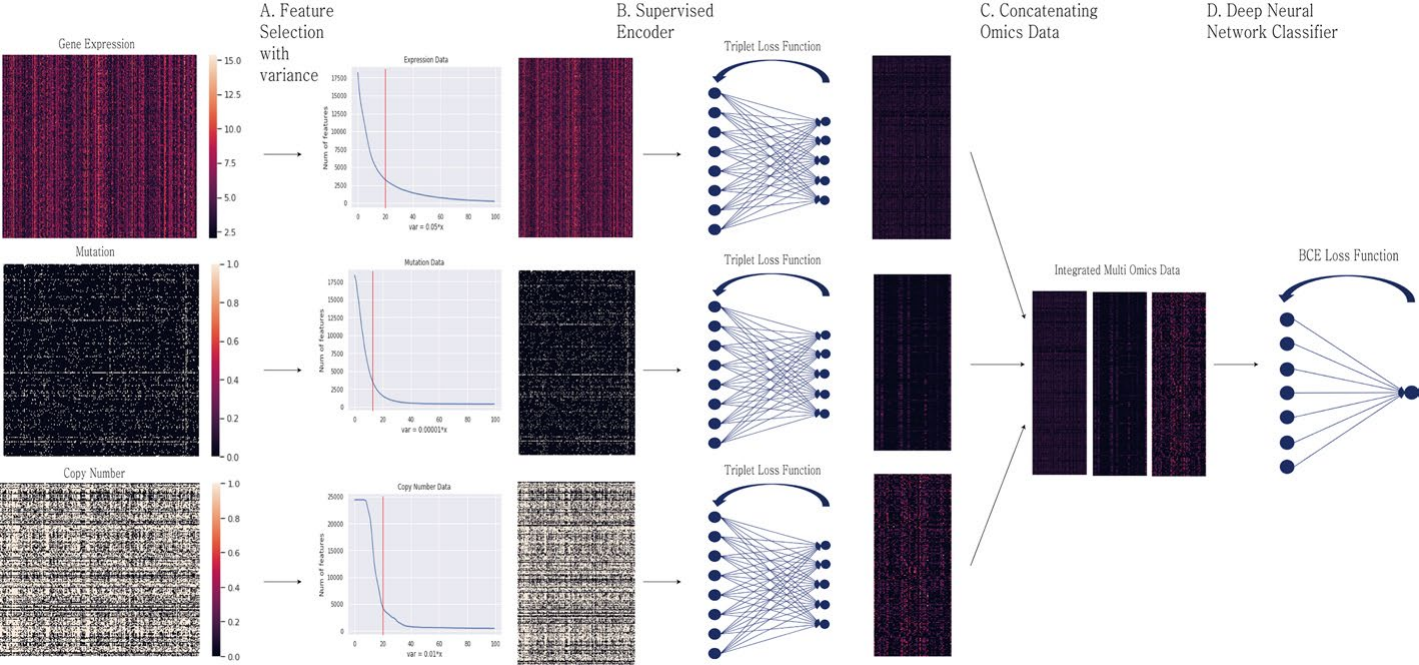
Workflow of Super.FELT. **a** Reduction of the large dimension of omics data with feature selection using a variance threshold based on the elbow method. **b** Supervised encoder using triplet loss function (SET) encodes each reduced omics data independently. **c** After encoding, all encoded omics data are integrated as the input data of the classifier. **d** A neural network classifier, for which the loss function is binary cross entropy (BCE) function, is trained for predicting drug response

Figure 1 shows the workflow of Super.FELT. It consists of i) feature selection using a variance threshold based on the elbow method, ii) a supervised encoder using triplet loss function (SET) for extracting important information from large-dimensional omics data, and iii) a classification based on a neural network for predicting drug response. Feature selection is important for improving the performance of SET, as large dimension is likely to cause overfitting. Each of the reduced omics dataset is independently encoded by SET, and the three encoded omics datasets are then concatenated as a single matrix. The integrated matrix is the input data of neural network for classification. The classifier should be simple to avoid overfitting because SET already transformed the omics data into a data representation optimized for drug response.

#### Feature selection and encoding

Gene expression, mutation, and CNA data are denoted as ${X_e,X_m,}$ and ${X_c}$, respectively. These data are in the form of ${N\times {M}}$ matrix, in which *N* is the number of samples and *M* is the number of features (*M* is different depending on data). We used triplet loss function, which helps the embedding vector to have similar values for samples with the same label. As the encoders were trained using the supervised mode, it should reduce the risk of overfitting. To decrease the risk, Super.FELT firstly reduced the dimension of omics data through feature selection using a variance threshold based on the elbow method. This was based on the assumption that genes with low variance might contain less important information. This approach had been used in several studies to handle omics data [37–39]. Second, we used dropout, weight decay, and early stopping. These techniques were frequently used to avoid overfitting. SET, the encoding function, has a single fully connected layer with a Rectified Linear Unit (ReLU) activation function to reduce the risk of overfitting.

In our study, feature selection using variance threshold for a certain omics data ${X_o}$ is denoted by ${F( X_o )}$.

1 $$\begin{aligned} X'_{o} = F(X_{o}), \end{aligned}$$

where ${X_o}$ is a ${N\times {M}}$ matrix and ${X'_o}$ is a ${N\times {M'}}$ matrix, ${M > M'}$. Thus, the reduced omics data, through feature selection using variance threshold, is denoted as ${X'_o}$.

Next, each omics dataset was assigned to an encoding function. Here, we defined the encoding function for the omics data as ${E_{O}(X'_o)}$.

2 $$\begin{aligned} r(X)&= {\left\{ \begin{array}{ll} X_{ij}, & X_{ij}\ge {0} \\ 0, & X_{ij} < 0 \end{array}\right. } \end{aligned}$$

3 $$\begin{aligned} E_{O}(X'_{o})&= r(X'_{o}W_{o}) \end{aligned}$$

4 $$\begin{aligned} {\tilde{X}}_o&= E_{O}(X'_{o}), \end{aligned}$$

where *r*(*X*) is the ReLU function, ${W_o}$ is a ${M'\times {{\tilde{M}}_{o}}}$ weight matrix, and ${{\tilde{X}}_o}$ is a ${N\times {{{\tilde{M}}_{o}}}}$ matrix. Encoding functions for gene expression, mutation, and CNA data are denoted as ${E_{E}(X'_{e})}$, ${E_{M} (X'_{m})}$, and ${E_{C}(X'_{c})}$, respectively. As each omics dataset had a different influence, the encoded data had different size. Among the omics data used in this study, gene expression was the most powerful to predict cancer drug response [17]. Although CNA and mutation are less influential than gene expression, they also contain important information and have been frequently used in many studies [13, 15, 25]. To utilize the property, we set the number of nodes of the encoders differently based on the importance of each omics dataset. Therefore, ${{\tilde{X}}_e= E_{E} (X'_{e})}$, ${{\tilde{X}}_m=E_{M}(X'_{m})}$, and ${{\tilde{X}}_c= E_{C}(X'_{c})}$ were ${N\times {{{\tilde{M}}_{e}}}}$, ${N\times {{{\tilde{M}}_{m}}}}$, and ${N\times {{{\tilde{M}}_{c}}}}$ matrices, respectively, and ${{\tilde{M}}_e>{\tilde{M}}_c>{\tilde{M}}_m}$. Note that ${\tilde{X}}_e$, ${\tilde{X}}_m$, and ${\tilde{X}}_c$ have continuous values after applying the encoding function. The final multi-omics data were generated by concatenating all omics matrices. The concatenating function is denoted as ${C({\tilde{X}}_e,{\tilde{X}}_m,{\tilde{X}}_c)}$.

5 $$\begin{aligned} C({\tilde{X}}_e,{\tilde{X}}_m,{\tilde{X}}_c)&= {\tilde{X}}_e\oplus {{\tilde{X}}_m}\oplus {{\tilde{X}}_c} \end{aligned}$$

6 $$\begin{aligned} {\hat{X}}&= C({\tilde{X}}_e,{\tilde{X}}_m,{\tilde{X}}_c), \end{aligned}$$

where ${\oplus }$ means the concatenation operator and ${{\hat{X}}}$ is the ${N\times {{\hat{M}}}}$ matrix, ${{\hat{M}}=({\tilde{M}}_e+{\tilde{M}}_m+{\tilde{M}}_c)}$.

For encoding omics data, SET uses triplet loss function [27] as a cost function. Triplet loss function makes the data with same label come closer than those with different labels. It requires three input objects, namely the anchor (baseline), positive, and negative. If we define an embedding function and Euclidean distance function as *f*(*x*) and *d*(*x*, *y*), respectively, the goal of triplet loss function is to ensure that the distance between anchor, positive, and negative is as follows.

7 $$\begin{aligned} d(f({\mathrm {a}}),f({\mathrm {p}}))\ll {d(f({\mathrm {a}}),f({\mathrm {n}}))}, \end{aligned}$$

where ${\mathrm {a}}$ is an anchor vector, ${\mathrm {p}}$ is a positive vector, and ${\mathrm {n}}$ is a negative vector. Therefore, the loss function is defined as:

8 $$\begin{aligned} {\mathcal {L}}_{T}({\mathrm {a,p,n}}))=max(d(f({\mathrm {a}}),f({\mathrm {p}}))-d(f({\mathrm {a}}),f({\mathrm {n}}))+\alpha ,0), \end{aligned}$$

where ${\alpha }$ is a margin between the positive and negative pair. The total loss of the embedding function would be:

9 $$\begin{aligned} {\mathcal {L}}_{T}(A, P, N)=\sum _{i=1}^K {\mathcal {L}}_{T, i}(A_i, P_i, N_i), \end{aligned}$$

where *A* is an anchor matrix, *P* is a positive matrix, and *N* is a negative matrix; ${A_i,P_i}$ and ${N_i}$ are i-th vector for each matrix, respectively, ${{\mathcal {L}}_{T, i}}$ is the loss for i-th pair sample, and *K* is the number of pairs.

Here, we had binary labels: resistant and sensitive. Therefore, when we select a sample with resistant label as the anchor, samples with resistant label become positive samples and those with sensitive label become negative samples and vice versa. For utilizing triplet loss function, we should define a pairing function, ${p(E_{O}(X'_{o}),\mathrm {y})}$, which makes all pairs for the two cases.

10 $$\begin{aligned}{} [X_{\mathrm {A}}, X_{\mathrm {P}}, X_{\mathrm {N}}]= \begin{bmatrix} \mathrm {x_S} &\quad \mathrm {x_S} &\quad \mathrm {x_R} \\ \mathrm {x_R} &\quad \mathrm {x_R} &\quad \mathrm {x_S} \\ \vdots &\quad \vdots &\quad \vdots \\ \mathrm {x_S} &\quad \mathrm {x_S} &\quad \mathrm {x_R} \\ \mathrm {x_S} &\quad \mathrm {x_S} &\quad \mathrm {x_R} \\ \end{bmatrix} =p(E_{O}(X'_{o}),\mathrm {y}) \end{aligned}$$

11 $$\begin{aligned} PE_{o}= \begin{bmatrix} PE_{o,1} \\ PE_{o,2} \\ \vdots \\ PE_{o,K-1}\\ PE_{o,K}\\ \end{bmatrix} =p({\tilde{X}}_{o},\mathrm {y}), \end{aligned}$$

where ${\mathrm {y}_{N\times {1}}}$ is a label vector for ${{\tilde{X}}_o, X_{\mathrm {A}}}$ is an anchor matrix, ${X_{\mathrm {P}}}$ is a positive matrix, ${X_{\mathrm {N}}}$ is a negative matrix, ${\mathrm {x_S}}$ and ${\mathrm {x_R}}$ are arbitrary row vectors of ${{\tilde{X}}_o}$ with sensitive label and resistant label, respectively, and ${PE_{o,i}}$ is an i-th pair sample for ${X_o}$. Therefore, the total loss of ${E_O}$ is expressed as:

12 $$\begin{aligned} {\mathcal {L}}_{T}(E_O(X'_o))=\sum _{i=1}^K {\mathcal {L}}_{T, i}(PE_{o,i}), \end{aligned}$$

#### Classification

The encoded data were already well organized to predict drug response owing to SET. As complex models pretend to  cause overfitting, the classifier should not be a complex model. Therefore, in our model, the classifier had a single layer with a sigmoid activation function, and dropout, weight decay, and early stopping were used. A classifier can be described as follows.

13 $$\begin{aligned} z={\hat{X}}\mathrm {w} \end{aligned}$$

14 $$\begin{aligned} s(z_j)&= {1\over {1+e^{-z_j}}} \end{aligned}$$

15 $$\begin{aligned} c({\hat{X}})&= s({\hat{X}}\mathrm {w}) \end{aligned}$$

16 $$\begin{aligned} \hat{\mathrm {y}}_{N\times {1}}&= c({\hat{X}}), \end{aligned}$$

where *s*(*x*) is a sigmoid function, ${{\hat{X}}}$ is a ${N\times {{\hat{M}}}}$ matrix, w is a ${{\hat{M}}\times {1}}$ weight vector, and ${\hat{\mathrm {y}}_{N\times {1}}}$ is a predicted label vector. The cost function is binary cross-entropy classification loss denoted by:

17 $$\begin{aligned} {\mathcal {L}}_{BCE}(c({\hat{X}}),{\mathrm {y}})=-[{\mathrm {y}}\log (c({\hat{X}}))+(1-{\mathrm {y}})\log (1-c({\hat{X}}))], \end{aligned}$$

where ${\mathrm {y}_{N\times {1}}}$ is the label vector.

## Results

To assess the performance of Super.FELT, we performed a series of experiments. In the experimental design, we introduced an overall experimental design of Super.FELT along with the other drug response prediction methods for comparison. We then assessed the performance of our model using four external datasets. The subsection ‘Cross-validation’ shows the results of ${5\times {5}}$-fold cross validation for 243 drugs in GDSC. In the subsection ‘On CCLE and CTRP’, we evaluated how well Super.FELT works on external cell line data. The subsection ‘On PDX and TCGA’ shows the translatability of Super.FELT from cell line data to non-cell line data.

### Experimental design

We evaluated Super.FELT with cross and external validation. As GDSC dataset included many cell lines and 243 drugs, we used it for training and cross validation. In cross validation using GDSC dataset, we employed ${5\times {5}}$-fold cross validation and generated validation data from 20% of training data, which meant that 20% was test data, 64% was training data, and 16% was validation data. In external validation, GDSC dataset was used for training, and CCLE, CTRP, PDX, and TCGA datasets were used for external test. For Super.FELT validation, we compared Super.FELT with the following  eight cases.

1. We compared with MOLI [25] because encoders and the classifier were trained jointly without feature selection in MOLI. By comparing Super.FELT with MOLI, we could identify that independent training with feature selection was better than joint training without feature selection.
2. We compared with MOLI using feature selection because we cannot say that independent training is better than joint training when comparing MOLI with Super.FELT. From the test, we could investigate which training was better and evaluate the effect of feature selection. We named this case 2 as MOLI after Feature selection (MOLIF).
3. We compared with a model using feature selection with variation threshold and an autoencoder followed by the neural network classifier; this was the same as Super.FELT, except that a latent space of the autoencoder was used for input of the classifier instead of SET. We could compare the simple autoencoder with SET. We named this case 3 as AE.
4. We compared with the model using feature selection and the neural network classifier; it was the same as Super.FELT without SET. Using this model, we examined how effective SET was. We named this case 4 as Artificial Neural Network after Feature selection (ANNF).
5. We compared with AutoBorutaRF [14], which is a random forest model after feature selection based on autoencoder and Boruta algorithm [26]. By testing AutoBorutaRF, we could know how effective the feature selection of Super.FELT and the classifier based on Artificial Neural Network are.
6. We compared with SVM after feature selection on gene expression data, which is similar to Huang et al. [11]. The difference is that Huang et al. [11] used a recursive feature elimination method [40, 41] for feature selection. However, the recursive feature elimination takes long time to eliminate unimportant features for predicting an output when the numbers of cell lines and drugs are large. Thus, instead of it, we used the same feature selection approach based on variance as Super.FELT. We named this case 6 as SVM.
7. We compared with Super.FELT using only gene expression without mutation and CNA data. Using this test, we could compare multi-omics approach by using gene expression alone. We named this case 7 as Super.FELT E.
8. We compared with Super.FELT using mutation and CNA data without gene expression. By testing this case 8, we could verify how influential mutation and CNA are on this task without gene expression. We named case 8 as Super.FELT M&C.

The GDSC dataset used in our experiment (Table 1) has an imbalanced label distribution, having a larger number of resistant samples. Thus, during training, we oversampled sensitivity samples based on the proportion of each label, except for case 5. In case 5, AutoBorutaRF [14] used an oversampling approach called EasyEnsemble [42], in which a predicted label was decided by the majority vote of random forest models trained on each balanced subset divided from a training dataset. In our comparison, the majority vote cannot be used because we evaluated models with AUC scores. Thus, we replaced the majority vote with the selection of the best model having the highest validation AUC score when testing AutoBorutaRF.

### Cross validation

**Table 5:** The average AUC scores for 243 drugs in GDSC for the cross validation test

	Super.FELT	MOLIF	AE	ANNF	MOLI	Super.FELT E	Super.FELT M&C	Auto BorutaRF	SVM
Validation AUC with set 1	0.727	0.711	0.712	0.694	0.715	0.723	0.602*		
Validation AUC with set 2	0.727	0.697	0.715*	0.699	0.71	0.726	0.598		
Validation AUC with set 3	0.728	0.698	0.69	0.697	0.704	0.726	0.597		
Validation AUC with set 4	0.73*	0.7137	0.707	0.696	0.694	0.73	0.592	0.747	0.702
Validation AUC with set 5	0.72	0.707	0.672	0.698	0.705	0.727	0.583		
Validation AUC with set 6	0.726	0.699	0.708	0.693	0.713	0.724	0.595		
Validation AUC with set 7	0.727	0.703	0.712	0.694	0.721*	0.715	0.597		
Validation AUC with set 8	0.727	0.7138*	0.684	0.703*	0.719	0.732*	0.593		
Test AUC	**0.729**	0.711	0.719	0.706	0.72	0.728	0.593	0.698	0.7

A bold value in the Test AUC indicates a method with the best performance

*bold values indicate the best validation AUC among eight hyperparameter sets

**Fig. 2:**
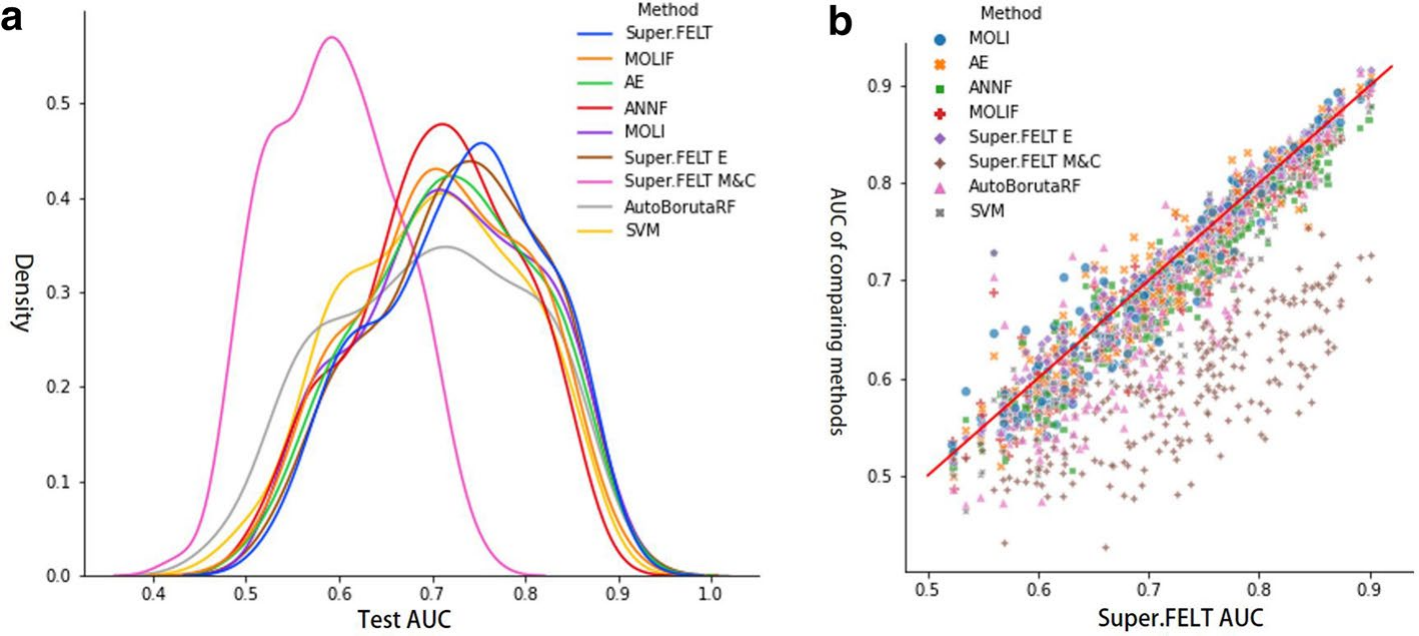
**a** The distributions of AUC values of cross validation on 243 drugs in GDSC for Super.FELT, MOLIF, AE, ANNF, MOLI, AutoBorutaRF, SVM, Super.FELT E, and Super.FELT M&C. **b** The scatter plot of cross validation AUC values, where *x*- and *y*-axis represent AUCs of Super.FELT and other methods, respectively

We performed ${5\times {5}}$-fold cross validation for 243 drugs in GDSC dataset. Additional file 1: Table S1 shows the profile of each drug. For hyperparameter tuning, we empirically constructed eight hyperparameter sets for Super.FELT, MOLIF, AE, and ANNF (Additional file 1: Tables S6 and S7). In the cases of Super.FELT E and M&C, we used same hyperparameter sets of Super.FELT. In MOLI, Sharifi-Noghabi et al. [25] did not report cross validation results of GDSC. Therefore, we constructed eight hyperparameter sets by randomly selecting candidate parameters, which were provided in the github of Sharifi-Noghabi et al. (Additional file 1: Tables S8 and S9). For AutoBorutaRF, we used all features provided by Xu et al. [14] and the same parameters as them. The validation set was used to select the hyperparameters set for test, based on the area under the average curve (AUC) values between true positives and false positives. Table 5 shows the average AUC values of 243 drugs for validation sets with eight hyperparameters and the test set, wherein the average test AUC values for Super.FELT, MOLIF, AE, ANNF, MOLI, AutoBorutaRF, SVM, Super.FELT E, and Super.FELT M&C were 0.729, 0.712, 0.719, 0.706, 0.720, 0.698, 0.7, 0.727, and 0.593, respectively (Additional file 1: Table S10). Figure 2A shows the distributions of AUC scores on 243 drugs for each method, and the distribution of Super.FELT is located on the right most among all methods. Although it seems that the difference in the distributions is small, the AUC values of Super.FELT were higher than those of other methods for most of the 243 drugs, with most dots (representing drugs) located under the diagonal (Fig. 2B).

**Fig. 3:**
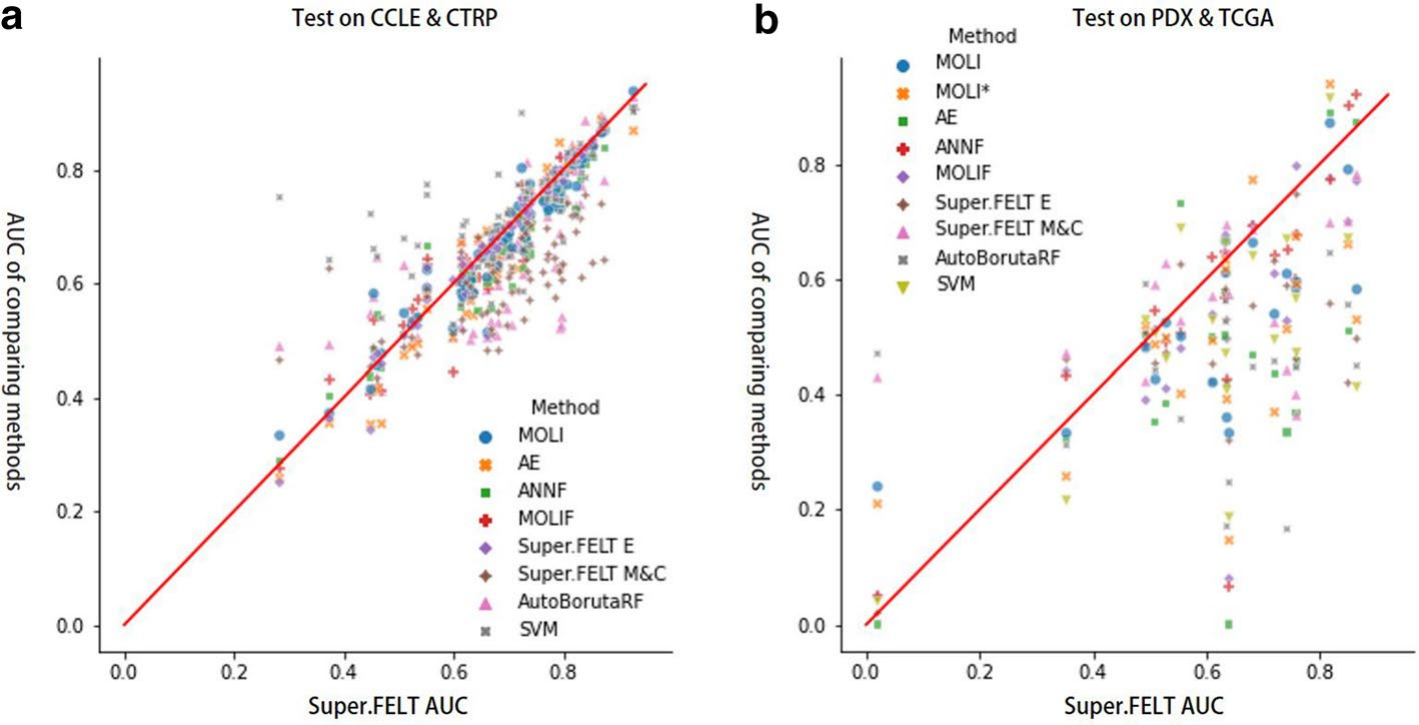
**a** The scatter plot of external validation AUC values on CTRP and CCLE. **b** The scatter plot of external AUC on PDX and TCGA. *x*- and *y*-axis represent AUCs of Super.FELT and other methods, respectively

### External validation

On external validation, GDSC was divided into five folds, where four folds and the remaining one were used for training and validation, respectively, and external datasets were used for the test. This process was repeated ${5\times {5}}$ times for every hyperparameter set, and we measured the average AUC values of external test when parameters with the highest average AUC values in the validation set were used. We used two different types of external datasets: cell line data (CCLE and CTRP) and non-cell line data (PDX and TCGA).

#### On CCLE and CTRP

In MOLI [25], they did not test on CCLE and CTRP. Therefore, we found hyperparameter sets using the same method as the subsection ‘Cross-validation’; however, hyperparameters can be different because they were randomly selected. For CCLE and CTRP, the average AUC scores for Super.FELT, MOLIF, AE, ANNF, MOLI, AutoBorutaRF, SVM, Super.FELT E, and Super.FELT M&C were 0.697, 0.685, 0.677, 0.674, 0.685, 0.663, 0.72, 0.693, and 0.607, respectively (Table 6 and Additional file 1: Tables S11, S12, S13, S14, S15, S16, S17, S18, and S19). Even though the average AUC of SVM is higher than others, Fig. 3A shows that Super.FELT obtained higher AUC scores than other methods for most drugs.

**Table 6:** Results of external validation on CCLE and CTRP

Drug	Super. FELT	MOLIF	AE	ANNF	MOLI	Super.FELT E	Super.FELT M&C	Auto BorutaRF	SVM
17-AAG(CCLE)	0.682	0.666	0.628	0.656	0.678	0.669	0.481	0.530	**0.717**
Afatinib(CTRP)	**0.798**	0.783	0.771	0.788	0.776	0.794	0.605	0.541	0.793
Axitinib(CTRP)	**0.795**	0.777	0.795	0.761	0.752	0.796	0.631	0.521	0.794
AZD7762(CTRP)	0.797	0.785	0.777	0.762	0.794	**0.800**	0.681	0.526	0.746
AZD8055(CTRP)	0.679	0.693	**0.698**	0.659	0.688	0.680	0.622	0.508	0.693
BI-2536(CTRP)	0.510	0.526	0.473	0.510	0.547	0.507	0.487	0.632	**0.678**
Bleomycin(CTRP)	0.631	0.602	0.569	0.614	0.605	**0.632**	0.594	0.500	0.574
BMS-345541(CTRP)	0.740	0.756	**0.759**	0.716	0.721	0.757	0.606	0.756	0.756
BMS-754807(CTRP)	0.671	0.665	**0.691**	0.649	0.662	0.677	0.510	0.666	0.690
Bortezomib(CTRP)	0.283	0.274	0.258	0.287	0.333	0.250	0.465	0.489	**0.751**
Bosutinib(CTRP)	**0.794**	0.778	0.773	0.760	0.730	0.791	0.624	0.688	0.727
CAL-101(CTRP)	**0.772**	0.748	0.748	0.724	0.734	0.764	0.642	0.751	0.748
Crizotinib(CCLE)	0.718	0.717	0.625	0.715	0.735	0.706	0.586	0.673	**0.773**
Cytarabine(CTRP)	0.831	0.810	0.830	0.781	0.809	0.834	0.713	0.739	**0.845**
Dabrafenib(CTRP)	0.840	0.824	0.832	0.810	0.821	0.838	0.755	**0.886**	0.785
Dasatinib(CTRP)	**0.669**	0.664	0.633	0.669	0.647	0.660	0.519	0.660	0.639
Docetaxel(CTRP)	0.469	0.412	0.354	0.450	0.476	0.458	0.497	0.412	**0.538**
Doxorubicin(CTRP)	**0.669**	0.634	0.606	0.555	0.654	0.655	0.631	0.532	0.563
Erlotinib(CCLE)	0.698	0.665	0.704	0.667	0.680	0.702	0.525	0.555	**0.722**
Etoposide(CTRP)	0.824	0.813	**0.827**	0.731	0.770	0.826	0.690	0.736	0.719
EX-527(CTRP)	0.739	0.706	0.721	0.700	**0.774**	0.724	0.570	0.764	0.747
GDC0941(CTRP)	0.704	0.702	**0.727**	0.693	0.723	0.699	0.618	0.651	0.648
Gefitinib(CTRP)	0.690	0.683	0.684	0.656	0.675	0.692	0.562	0.649	**0.698**
Gemcitabine(CTRP)	0.614	0.598	**0.671**	0.557	0.583	0.644	0.655	0.523	0.562
GW843682X(CTRP)	0.454	0.534	0.413	0.483	0.582	0.469	0.486	0.576	**0.660**
Imatinib(CTRP)	0.682	0.679	0.648	0.690	0.698	0.691	0.549	0.596	**0.791**
JNJ-26854165(CTRP)	0.740	0.707	0.698	0.650	**0.745**	0.739	0.701	0.671	0.667
KU-55933(CTRP)	0.653	0.661	0.646	0.650	0.647	**0.665**	0.544	**0.665**	0.639
Lapatinib(CTRP)	0.661	0.638	**0.692**	0.662	0.610	0.656	0.548	0.588	0.688
Masitinib(CTRP)	0.820	0.813	0.816	0.821	0.809	**0.822**	0.721	0.807	0.815
Methotrexate(CTRP)	0.729	0.722	0.724	0.730	0.699	**0.746**	0.535	0.684	0.762
MG-132(CTRP)	0.374	0.433	0.355	0.403	0.372	0.363	0.626	0.492	**0.641**
Mitomycin C(CTRP)	**0.707**	0.655	0.627	0.593	0.662	0.699	0.584	0.526	0.627
MK-2206(CTRP)	0.733	0.733	0.710	0.717	0.742	0.733	0.642	**0.754**	0.753
Nilotinib(CCLE)	0.616	0.577	0.638	0.583	0.601	0.613	0.535	0.675	**0.683**
Nutlin-3a(CCLE)	0.928	0.906	0.868	0.910	**0.938**	0.908	0.906	0.928	0.902
NVP-BEZ235(CTRP)	**0.636**	0.584	0.543	0.607	0.583	0.626	0.576	0.512	0.616
NVP-TAE684(CTRP)	0.535	0.571	0.494	0.538	0.540	0.525	0.474	0.505	**0.666**
Obatoclax Mesylate(CTRP)	**0.672**	0.613	0.618	0.607	0.671	0.666	0.619	0.611	0.644
OSI-027(CTRP)	0.701	0.690	0.698	0.674	0.688	**0.702**	0.610	0.684	0.646
OSI-930(CTRP)	0.808	0.795	0.806	0.819	0.773	0.824	0.740	0.747	**0.829**
PAC-1(CTRP)	0.734	0.660	0.706	0.661	0.729	0.713	0.706	**0.812**	0.760
Paclitaxel(CTRP)	0.449	0.405	0.352	0.436	0.414	0.343	0.453	0.547	**0.722**
Parthenolide(CTRP)	0.623	0.642	0.546	0.616	0.592	0.580	0.601	0.609	**0.708**
Pazopanib(CTRP)	**0.680**	0.635	0.616	0.659	0.631	0.661	0.602	0.651	0.679
PD-0325901(CCLE)	**0.854**	0.841	0.843	0.821	0.840	0.852	0.635	0.828	0.826
PD-0332991(CCLE)	0.645	0.609	0.678	0.552	0.651	0.645	**0.680**	0.676	0.629
PHA-665752(CCLE)	0.524	0.553	0.487	0.535	0.527	0.534	0.504	0.495	**0.612**
PHA-793887(CTRP)	0.822	0.808	**0.835**	0.818	0.815	0.813	0.720	0.798	0.826
PI-103(CTRP)	**0.785**	0.759	0.758	0.742	0.734	0.751	0.643	0.750	0.781
PIK-93(CTRP)	0.817	0.821	0.826	0.807	0.828	0.820	0.718	**0.844**	0.814
piperlongumine(CTRP)	0.727	0.639	0.682	0.627	0.657	**0.740**	0.658	0.616	0.585
PLX4720(CCLE)	0.875	0.873	0.871	0.839	0.868	**0.884**	0.640	0.781	0.870
Ruxolitinib(CTRP)	0.763	0.756	0.775	0.745	0.744	0.773	0.619	0.769	**0.777**
SN-38(CTRP)	0.735	0.730	0.717	0.675	0.749	0.721	0.620	0.742	**0.776**
SNX-2112(CTRP)	**0.800**	0.789	0.782	0.740	0.793	0.779	0.633	0.759	0.672
Sorafenib(CCLE)	0.552	0.633	0.555	0.666	0.623	0.627	0.576	0.595	**0.773**
Sunitinib(CTRP)	0.552	0.642	0.578	0.628	0.591	0.570	0.589	0.580	**0.755**
Tamoxifen(CTRP)	0.617	0.630	0.591	0.619	0.575	0.632	0.516	0.609	**0.701**
Temozolomide(CTRP)	**0.834**	0.827	0.820	0.826	0.818	0.825	0.609	0.771	0.794
Temsirolimus(CTRP)	0.731	0.692	0.689	0.654	0.679	0.726	0.688	**0.762**	0.748
TG101348(CTRP)	0.771	0.786	**0.803**	0.754	0.774	0.770	0.696	0.790	0.781
TGX221(CTRP)	0.461	0.421	0.414	0.544	0.455	0.476	0.434	**0.646**	**0.646**
TPCA-1(CTRP)	0.784	0.779	0.788	0.778	**0.795**	0.777	0.668	0.793	**0.795**
Trametinib(CTRP)	0.772	**0.783**	0.746	0.772	0.770	0.761	0.597	0.717	0.773
Tubastatin A(CTRP)	0.870	0.866	0.888	0.865	0.865	0.872	0.739	**0.895**	0.878
TW 37(CTRP)	0.599	0.444	0.504	0.512	0.520	**0.606**	0.511	0.505	0.527
Vorinostat(CTRP)	0.793	0.820	**0.847**	0.776	0.749	0.799	0.619	0.826	0.745
VX-680(CTRP)	0.724	0.773	0.719	0.715	0.802	0.749	0.679	0.750	**0.899**
YK 4-279(CTRP)	0.786	0.763	0.785	0.759	0.765	**0.800**	0.574	0.628	0.747
YM155(CTRP)	**0.662**	0.591	0.602	0.601	0.513	0.507	0.481	0.505	0.632
ZSTK474(CTRP)	0.773	**0.783**	0.779	0.756	0.729	0.771	0.651	0.753	0.770
AVG	0.697	0.685	0.677	0.674	0.685	0.693	0.607	0.663	**0.721**

Bold values indicate methods with best performance for each drug

**Table 7:** Results of external validation on PDX and TCGA

Drug	Super. FELT	MOLIF	AE	ANNF	MOLI	Super.FELT E	Super. FELT M&C	MOLI*	Auto BorutaRF	SVM
5-Fluorouracil(PDX)	0.865	0.77	0.872	**0.919**	0.582	0.496	0.782	0.529	0.449	0.413
5-Fluorouracil(TCGA)	0.493	0.390	0.502	0.486	0.481	0.506	0.423	0.519	**0.591**	0.528
Cetuximab(PDX)	0.636	0.496	0.42	0.426	0.360	0.529	**0.694**	0.392	0.171	0.407
Cetuximab(TCGA)	0.353	0.440	0.324	0.433	0.333	0.460	**0.471**	0.258	0.312	0.216
Cisplatin(TCGA)	0.759	**0.796**	0.459	0.678	0.595	0.747	0.364	0.590	0.449	0.471
Docetaxel(TCGA)	0.611	0.538	0.498	**0.637**	0.421	0.452	0.570	0.493	0.422	0.528
Doxorubicin(TCGA)	0.510	0.512	0.352	0.545	0.426	0.453	**0.590**	0.486	0.441	0.505
Erlotinib(PDX)	**0.742**	0.527	0.334	0.652	0.609	0.588	0.442	0.513	0.166	0.669
Erlotinib(TCGA)	0.85	0.7	0.51	**0.9**	0.790	0.420	0.700	0.660	0.555	0.670
Etoposide(TCGA)	0.020	0.050	0.000	0.050	0.240	0.020	0.430	0.210	**0.470**	0.040
Gemcitabine(PDX)	0.682	0.694	0.468	0.691	0.663	0.553	0.686	**0.772**	0.447	0.640
Gemcitabine(TCGA)	**0.634**	0.575	0.501	0.568	0.611	0.582	0.613	0.616	0.526	0.470
Mitomycin C(TCGA)	**0.640**	0.080	0.000	0.067	0.333	0.320	0.573	0.147	0.247	0.187
Paclitaxel(PDX)	**0.72**	0.609	0.436	0.642	0.539	0.631	0.525	0.369	0.457	0.494
Paclitaxel(TCGA)	0.529	0.41	0.383	0.49	0.524	0.472	**0.627**	0.497	0.498	0.461
Sorafenib(TCGA)	0.818	0.775	0.889	0.772	0.871	0.557	0.698	**0.938**	0.645	0.914
Tamoxifen(TCGA)	**0.758**	0.679	0.368	0.6	0.584	0.461	0.400	0.674	0.446	0.566
Temozolomide(TCGA)	0.634	**0.676**	0.578	0.649	0.666	0.643	0.649	0.633	0.562	0.662
Trametinib(PDX)	0.555	0.479	**0.732**	0.506	0.5	0.625	0.527	0.404	0.357	0.688
AVG	**0.622**	0.537	0.454	0.564	0.533	0.501	0.567	0.511	0.432	0.501

Bold values indicate methods with best performance for each drug

MOLI* was the test AUC of randomly selecting hyperparameters having best validation.

MOLI was tuned using hyperparameters from Additional file 1: Table S3 of Sharifi-Noghabi et al. [25]

#### On PDX and TCGA

**Fig. 4:**
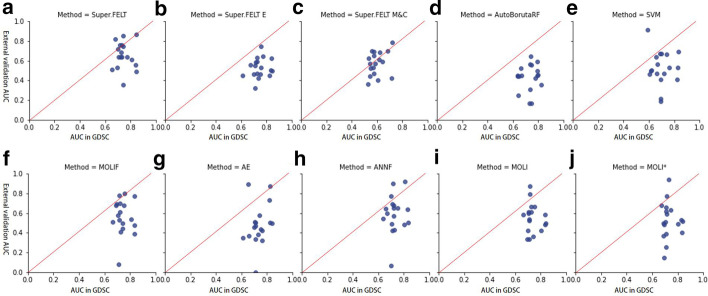
Comparison between the AUC values in GDSC and the test AUC values of drugs on PDX and TCGA for **a** Super.FELF, **b** Super.FELF E, **c** Super.FELF M&C, **d** AutoBorutaRF, **e** SVM, **f** MOLF, **g** AE, **h** ANNF, **i** MOLI, and **j** MOLI*

Although MOLI also performed external validation, datasets used in this study are different from those used in MOLI because our datasets contain more drugs than those used by Sharifi-Noghabi et al. [25]. Even for the same drugs, the number of samples on training and test data would be different. For selecting hyperparameters in MOLI, we used the same hyperparameters available from Supplementary materials of Sharifi-Noghabi et al. [25] (Additional file 1: Tables S20, S21, S22, S23, S24, S25, and S26). However, for some drugs not tested in MOLI, if data were obtained from TCGA, we used the hyperparameters of “Cisplatin” because it has the highest AUC value in the TCGA dataset. For PDX dataset, although AUC of “Paclitaxel” was higher than that of “Gemcitabine” in Sharifi-Noghabi et al [25], the latter was the best case in our experiment. Therefore, if we tested drugs of PDX for external validation, hyperparameters of “Gemcitabine” were used. In addition, we tested in MOLI using new hyperparameter sets created by the same method as the subsection ‘On CCLE and CTRP’ because our data would be different from the data used by Sharifi-Noghabi et al. [25]. We called this case as MOLI*. Table 7 shows that the average AUC scores for Super.FELT, MOLIF, AE, ANNF, Super.FELT E, Super.FELT M&C, MOLI, AutoBorutaRF, SVM, and MOLI* were 0.622, 0.537, 0.454, 0.564, 0.501, 0.567, 0.533, 0.432, 501, and 0.511, respectively (Additional file 1: Tables S27, S28, S29, S30, S31, S32, S33, S34, S35, and S36). Note that Etoposide (TCGA) is hard to predict the drug response, showing that the average AUC score of all methods is 0.152. Figure 3B shows that the performance of Super.FELT is superior to that of other methods for most drugs. In addition, when we compared the AUC values in GDSC and the test AUC values on PDX and TCGA for each drug, we could observe that Super.FELT showed high AUC values for both internal and external validation (Fig. 4).

**Fig. 5:**
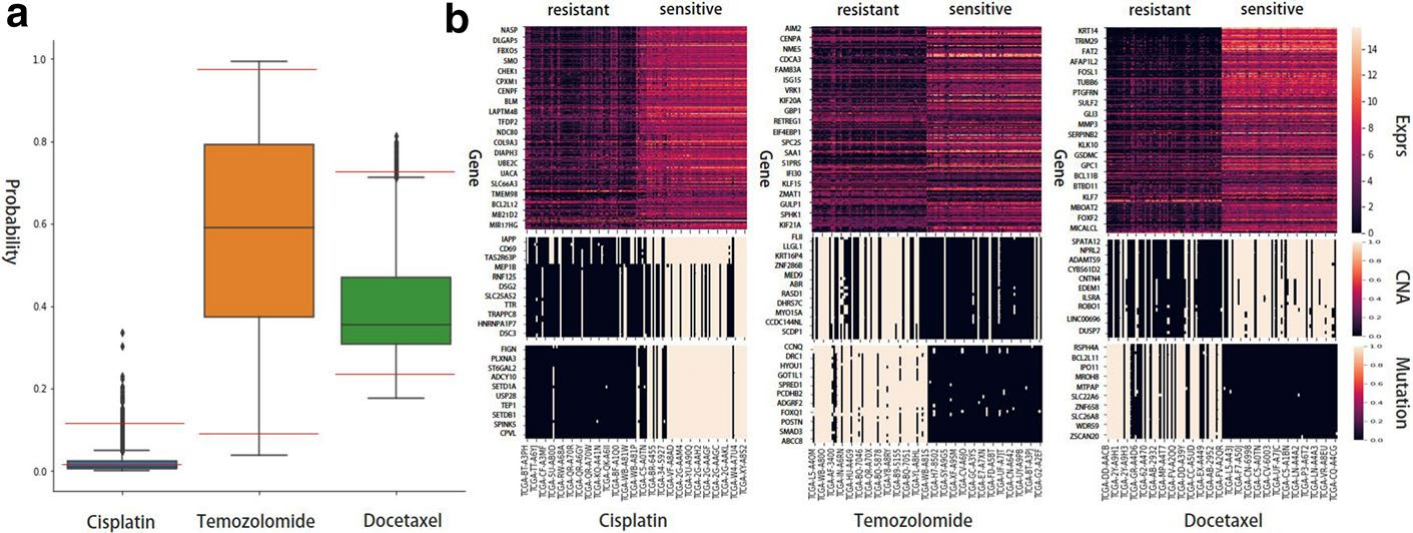
**a** The box plots of drug response prediction probability of Cisplatin, Temozolomide, and Docetaxel on TCGA samples, respectively. The red lines represent the top and bottom 1% prediction probabilities on samples. **b** Heat maps of gene expression, CNA, and mutation values of most discriminative genes between resistant and sensitive samples of Cisplatin, Temozolomide, and Docetaxel

### Pharmacogenomics analysis

The in vivo drug response data such as TCGA are important to understand the pharmacogenomics of drugs, but the available in vivo drug response data are small. However, TCGA contains multi-omics data for thousands of samples without drug response data. Thus, we predicted drug response of the samples in TCGA using our model trained with GDSC and investigated most contributing genomic features related to drug response, which is similar to the analysis performed by Chiu et al. [43]. For 6194 TCGA samples having gene expression, CNA, and mutation data, we calculated the drug response probability on cisplatin, temozolomide, and docetaxel using Super.FELT. Then, for each drug, we constructed two groups of samples, 1% of the most sensitive samples and 1% of the most resistant samples (Fig. 5A). We selected 200, 50, and 50 genes with the most different values between the two groups in gene expression, CNA, and mutation data, respectively, using a *t*-test. These genes might be contributing genomic features for the drug response. The genes related to cisplatin, temozolomide, and docetaxel are shown in Additional file 1: Tables S37, S38, and S39, respectively, and Fig. 5B shows heat maps of each omics data. For these genes, we performed a functional enrichment test using the Database for Annotation, Visualization and Integrated Discovery [44] for gene ontology (GO) terms [45].

Cisplatin is an alkylating agent used to treat a number of cancers. The alkylating agent inhibits DNA synthesis and RNA transcription by damaging DNA [46]. The functional enrichment test on genes related to cisplatin showed that cell division (GO:0051301), DNA replication (GO:0006260), and cellular response to DNA damage stimulus (GO:0006974) are enriched, with a false discovery rate (FDR) [47] of 6.13E-13, 1.54E-08, and 0.00244, respectively. It is supporting that the genes discriminating the sensitive and resistant samples are related to DNA synthesis. The enrichment results on GO biological process are shown in Additional file 1: Table S40.

Temozolomide is an alkylating agent used in the treatment of brain tumors. It methylates the purine bases of DNA and the affected DNA triggers the death of tumor cells [48]. From the enrichment test, we found significant terms related with temozolomide mechanisms including mitotic nuclear division (GO:0007067), cell division (GO:0051301), and DNA replication (GO:0006260), where their FDRs are 1.73E-10, 1.73E-10, and 7.59E-06, respectively (Additional file 1: Table S41).

Docetaxel, a taxoid antineoplastic agent, binds to microtubules and inhibits depolymerization of microtubules induced by calcium ions [49]. Thus, it disrupts the cytoskeleton of malignant cells during the mitotic phase [50]. We found significant molecular function terms of GO including calcium ion binding (GO:0005509) and structural constituent of cytoskeleton (GO:0005200) with FDRs of 0.00135 and 0.00135, respectively (Additional file 1: Table S42).

## Discussion

In drug response prediction, the translatability from cell line data to non-cell line data is important because non-cell line data, such as PDX and TCGA, are of high cost and have a small number of samples. Thus, we often rely on cell line data, such as GDSC, CCLE and CTRP, to train machine-learning models. However, the model trained on cell line data tends to not work well for non-cell line data owing to various elements, such as the high dimension of omics data and its batch effects. Previous studies to predict drug response have frequently focused on reducing the large omics dimension. In particular, AE was mostly used in various models [12–16].

In the section ‘Cross-validation’, the average AUCs of all drugs for each method were greater than 0.69, except for Super.FELT M&C. This indicates that gene expression is the most important data for this task, and it is difficult to obtain a distinct difference on the results of methods on cross validation test when the input data includes gene expression, in terms of the average AUC score. However, as shown in Fig. 2B, Super.FELT showed higher scores than the other methods in most of the cases.

The aspect of results in the section ‘On CCLE and CTRP’ was similar to that in the section ‘Cross-validation’ except that SVM achieved the best performance in terms of the average score. The result showed that we could obtain high translatability just by normalizing between two different platform types of gene expression.

In the section ‘On PDX and TCGA’, AE and AutoBorutaRF were the worst among the models unlike the section ‘Cross-validation’ and ‘On CCLE and CTRP’. Although previous studies frequently used AE [12–16], the model based on AE could not obtain good performance for the external non-cell line data in our experiment. As the embedded data of AE, an unsupervised training model, were not encoded to predict drug response, AE trained on cell line and feature selection based on AE would not be proper for external non-cell line data. In contrast, although Super.FELT, MOLIF, and MOLI were trained using GDSC dataset, those showed better performance than AE. The reason is that the encoder using triplet loss function was trained for focusing on the features that decide drug response. This indicates that although test data have different properties from training data, the encoder using triplet loss function could learn the important features for determining the drug response. From these results, we could estimate that AE could not distinguish dataset-specific features and biological features, and it could not extract proper biological features for external data.

For evaluating feature selection using variance thresholds based on the elbow method, the results of ANNF, MOLIF, and MOLI in the section ‘On PDX and TCGA’ seem to be useful. First, although ANNF consists of feature selection and the simple classifier, it provided the better results than MOLIF and MOLI. Second, comparison of MOLIF and MOLI revealed that feature selection also seems to improve the performance of the encoder. Given these results, we suggest that feature selection is powerful for external validation of different types of data.

By comparing Super.FELT with MOLIF, based on ANNF, in the section ‘On PDX and TCGA’, we verified the importance of independent training. Although both Super.FELT and MOLIF used feature selection and triplet loss function, their results were better and inferior than that of ANNF, respectively. This shows that it is helpful to not use encoders jointly trained with the classifier.

From the perspective of omics data, the performance of Super.FELT M&C was impressive on PDX and TCGA dataset. Although Super.FELT M&C did not use gene expression data, the result of Super.FELT M&C was the second best. In Cetuximab (TCGA), Doxorubicin (TCGA), Paclitaxel (TCGA), and Cetuximab (PDX), Super.FELT M&C showed the best performance. When comparing Fig. 4C with other plots in Fig. 4, the AUCs of Super.FELT M&C in GDSC were most positively correlated with those in PDX and TCGA, although its average AUC was lower than that of Super.FELT. Therefore, we could confirm the importance of mutation and CNA data for the translatability to non-cell line data.

Because of its properties, Super.FELT, which used feature selection, encoder using triplet loss, independent training, and multi omics approach, outperformed the other methods with the average AUC value of 0.622 in the section ‘On PDX and TCGA’. In case of drugs for which Super.FELT was not the best, AUC values of Super.FELT were similar to those of the best model, except the case of Etoposide (TCGA) (Fig. 3B).

Recent studies have proposed models with adversarial networks by training both in vitro and in vivo datasets and obtained increased performance compared with that of models trained using only in vitro datasets [51]. This approach is helpful when appropriate in vivo datasets for a given drug are available. However, when there is no in vivo dataset available for a given drug, our proposed Super.FELT model can be used to predict drug response in patients.

In this study, although Super.FELT was applied for the drug response prediction, it can be further applied for other biomedical tasks using multi-omics datasets. Recently, disease progress prediction, such as survival and recurrence, and cancer subtype classification,  has been performed using multi-omics datasets [52–55]. In those studies, AE, a chi-squared test, and a feedforward network have been used to represent features in omics data. Super.FELT can further improve prediction by applying the supervised learning approach for feature representation.

## Conclusion

In our study, we found that high translatability could be achieved between the same cell line data, but it is difficult to achieve reasonable translatability from cell line data to non-cell line data. To achieve high translatability, we used feature selection using variance thresholds based on the elbow method and triplet loss function, which are often used for improving the encoder in image classification models [56, 57]. Our study focused on how to properly utilize triplet loss function unlike MOLI [25]. From the result of the case that ANNF afforded higher scores than MOLI and MOLIF in the section ‘On PDX and TCGA’, it can be suggested that using triplet loss function improperly is worse than not using it. Additionally, we reported the strength of feature selection using a variance threshold based on the elbow method. The translatability of Super.FELT was found to be quite high because the average AUC of internal cross validation test, external validation on cell line data, and non-cell line data were 0.729, 0.697, and 0.622, respectively.

## Supplementary Information

**Additional file 1**. Supplementary Tables.

## Data Availability

The datasets generated and analyzed during the current study are available in the Super.FELT repository: http://github.com/DMCB-GIST/Super.FELT.

## Abbreviations

CNA: Copy number aberration
SET: Supervised encoder using Triplet loss function
ANNF: Artificial neural network after feature selection
MOLIF: MOLI after feature selection
AE: Auto encoder
Super.FELT E: Super.FELT using gene expression data
Super.FELT M[MYAMPC]: Super.FELT using mutation and CNA data
